# Return-to-Work Within a Complex and Dynamic Organizational Work Disability System

**DOI:** 10.1007/s10926-015-9613-2

**Published:** 2015-11-07

**Authors:** Arif Jetha, Glenn Pransky, Jon Fish, Lawrence J. Hettinger

**Affiliations:** 1 Liberty Mutual Research Institute for Safety, 71 Frankland Road, Hopkinton, MA 01748 USA; 2 Department of Work Environment, University of Massachusetts-Lowell, One University Avenue, Lowell, MA 01854 USA

**Keywords:** Return-to-work, Sociotechnical systems, System dynamics modeling, Complexity, Work disability management

## Abstract

*Background* Return-to-work (RTW) within a complex organizational system can be associated with suboptimal outcomes. *Purpose* To apply a sociotechnical systems perspective to investigate complexity in RTW; to utilize system dynamics modeling (SDM) to examine how feedback relationships between individual, psychosocial, and organizational factors make up the work disability system and influence RTW. *Methods* SDMs were developed within two companies. Thirty stakeholders including senior managers, and frontline supervisors and workers participated in model building sessions. Participants were asked questions that elicited information about the structure of the work disability system and were translated into feedback loops. To parameterize the model, participants were asked to estimate the shape and magnitude of the relationship between key model components. Data from published literature were also accessed to supplement participant estimates. Data were entered into a model created in the software program Vensim. Simulations were conducted to examine how financial incentives and light duty work disability-related policies, utilized by the participating companies, influenced RTW likelihood and preparedness. *Results* The SDMs were multidimensional, including individual attitudinal characteristics, health factors, and organizational components. Among the causal pathways uncovered, psychosocial components including workplace social support, supervisor and co-worker pressure, and supervisor-frontline worker communication impacted RTW likelihood and preparedness. Interestingly, SDM simulations showed that work disability-related policies in both companies resulted in a diminishing or opposing impact on RTW preparedness and likelihood. *Conclusion* SDM provides a novel systems view of RTW. Policy and psychosocial component relationships within the system have important implications for RTW, and may contribute to unanticipated outcomes.

## Background

Returning to work after an occupational injury or illness can be a complex process. In an ideal scenario, injured workers follow a uniform return-to-work (RTW) trajectory that consists of a series of evolving phases including seeking medical care, recovery and sustained work re-entry [1]. In many cases, however, RTW is not a linear process, and a proportion of injured workers experience a variable and often undesirable RTW course including extended (e.g., staying out of work for a longer than expected period of time) or intermittent work disability (e.g., a person alternates between being able to perform work tasks and absenteeism) that results in significant individual, employer, and societal costs [2, 3]. Complexity in organizational work disability systems might be a source of variability and adverse RTW outcomes, and may explain why employer-based work disability management strategies do not always have the intended effect of benefiting RTW. To address complexity in RTW, we apply sociotechnical systems thinking and utilize system dynamics modeling (SDM) to develop new insights on how multiple influential factors make up the work disability system and impact RTW. Findings from this study have important implications for advancing our understanding of RTW, and the design of employer-based work disability management strategies.

Within the context of contemporary biopsychosocial models of work disability [4–6], studies have generated an understanding of components that influence the RTW process and define the work disability system. Influential components include health factors (e.g., injury or illness severity, activity limitations, characteristics of symptoms, and rate of recovery) [7, 8], worker perceptions (e.g., readiness to return to work, and recovery expectations) [7–10], management and co-worker relationships (e.g., workplace social support, and communication) [8, 11–13], physical and psychosocial job demands [11], availability of modified duties or job accommodations [7, 14, 15], and access to health care [7]. However, existing work disability models do not provide detailed quantitative descriptions of how these components dynamically interact with one another over time. Most studies have assumed a linear and constant relationship among components that influence RTW and provide a potentially oversimplified perspective of the dynamic process [16].

Scientists in the field of work disability prevention are increasingly discussing the need to take a systems-focused view towards examining complexity [2, 6, 12]. In their qualitative study, MacEachen et al. [2] suggest that complexity in organizations may contribute to undesirable RTW outcomes. In particular, the authors posit that extended sickness absence may be caused by highly interrelated components within individual, workplace, health care, vocational retraining, and workers compensation subsystems. The nature of each component can cumulatively contribute to variability in RTW. Examples include the opposing goals of employers (e.g., minimize costs) and workers (e.g., recovery from illness), a lack of coordination and communication among work disability stakeholders (e.g., workers, health care providers, employers, and workers compensation insurers), and gaps in the provision of adequate accommodations [2]. Within the perspective of a system of interrelated components, both minor administrative responsibilities and company-wide work disability management policies can have ripple effects across the system as a whole, resulting in suboptimal RTW outcomes. Other qualitative research describes the workplace as a social system in which the relationships between various actors can influence RTW [13, 17, 18]. In these studies, supervisors and co-workers were often responsible for managing different phases of the RTW process and their interaction with injured workers played an important role in determining work disability outcomes. Yet, social relations were not always considered when designing and implementing work disability-related policies, reducing the successfulness and sustainability of RTW [17].

Building on qualitative findings, researchers may draw on sociotechnical systems theory which considers organizations as complex adaptive systems made up of interdependent personal, social, technical and organizational components that interact with one another in frequently non-linear ways, and thus can make processes like RTW less uniform [19, 20]. By applying a sociotechnical systems perspective to work disability research, feedback relationships among system components can be examined to understand the underlying causes of RTW outcomes [21]. System dynamics modeling (SDM) is one specific methodology that can be used to depict and simulate the activity of complex systems. SDM utilizes feedback loops to describe the functional relationships among components and can signify an amplifying (e.g. action generating) or balancing (e.g. maintaining status quo or dampening) effect on outcome variables of interest within the modeled system [19]. Through stakeholder-based estimates of component relations, SDM also involves generating a simulation tool to test dynamic hypotheses, and quantify the system-wide effect of modifying different variables. SDM was initially designed for understanding finite systems in the fields of business and engineering [19]. A more recent application has been to understand challenging public health problems [22–24] where scientists uncovered aspects of the system that could be amenable to intervention, and relatively small changes might lead to significantly improved systemic outcomes. By taking a sociotechnical systems perspective and utilizing SDM methodology researchers and practitioners may also be able to better understand the broader impacts of work disability management policies or programs on RTW.

This study describes a novel application of a sociotechnical systems perspective to better understand the RTW process. In particular, SDM was used to identify and model complex non-linear relationships among key components of organizational work disability systems, and generate quantitative simulations to understand how varying work disability-related policies might impact the likelihood of successful RTW.

## Methods

A multi-staged model building methodology was applied to develop the organizational SDM and will be summarized in the following sections [25]. An in-depth description of the model building methodologies, and piloting procedure can be accessed from a previously published article [25]. The study protocol was approved by the New England Institutional Review Board (Protocol # 14-189).

### Participating Companies and Recruitment

Models were developed within two companies in the United States that reported having complex RTW problems. The first was a food manufacturing company, and second was an industrial service company. Despite their differences, the types of job demands performed by frontline workers (e.g., moderate-to-heavy lifting, and fast pace), and organizational structures were similar.

Stakeholders representing various positions across each company’s organizational hierarchy were invited to participate. To be eligible, participants had to be fluent in English, over the age of 18 years, and have direct or indirect experience with the RTW process. Each company provided a list of eligible participants to the investigators. Potential participants were contacted via email or phone and invited to participate. Individuals who agreed to participate in model building received lunch for their involvement. A total of 30 stakeholders took part in model building, 15 from each company. Participants included senior managers (e.g., human resource manager, health and safety coordinator, workers compensation coordinator, financial service manager; n = 10), frontline supervisors (n = 10), and frontline workers (n = 10).

### Model Construction Process

Participants engaged in two to three iterative model building sessions that were conducted in one-on-one (senior managers) or focus group-style formats (frontline workers or frontline supervisors). Each session lasted between 60 and 90 min, and occurred over the participant’s lunch break. Overall, participant time commitment ranged from 2 to 3 h. During model building sessions, diverse perspectives regarding the RTW process within each organization and integrated insights were collected and incorporated into a representative SDM [25]. To minimize social desirability biases and encourage critical conversations, model building occurred separately with each group of participants.

Model builders facilitated discussions that elicited the structure and process of the work disability system in each company, while concurrently translating conversations into visual mapping of the SDM [26]. Questions asked during model building sessions followed a similar format in each company [25]. Participants were first asked to describe the general RTW process in their organization. Then specific aspects of the RTW process that respondents reported as important were probed. When participants discussed an influential component, follow-up questions asked about how the component might influence RTW and its relationship to other components in the system [25]. The model boundary was set at the organizational level to provide focus to the sessions, and enable comparisons between the two companies that were located in different contexts. Accordingly, model-building questions were framed to uncover components within the organizational boundary of the system. When community-, state-, or federal-level factors were discussed, they were categorized as exogenous and not included in the final model.

Components uncovered through interviews and focus groups were clustered into common themes and translated into dynamic feedback loops by the research team. All feedback loops were described to study participants who were asked for their level of agreement regarding their accuracy in representing the actual process. In cases where there was disagreement, follow-up questions were posed to encourage participants to think critically about the structure of the model. Based on the responses to the additional questions, the model was iteratively refined until stakeholder agreement on the description of each feedback loop was reached [25].

### Parameterization and Simulation

After determining the feedback structure of the SDM, participants were presented with axes that included a system component on the horizontal axis, and an outcome of the feedback loop (e.g., RTW preparedness) on the vertical axis. Participants were asked to estimate the direction, shape, and magnitude of the relationships between the variables on each axis, forming a reference mode [25]. As participants described the relationship between the components, the model builder would draw the reference mode on chart paper. Subsequently, the reference mode figure was presented and described to study participants to confirm whether it reflected their perceptions [25]. Based on the final shape of the reference mode, a differential equation was generated. Data from published literature were also accessed to supplement participant estimates.

All qualitative and quantitative data collected from model building was entered into the software program Vensim [27]. In addition to capturing the structure of the system, Vensim also enabled simulation capabilities to test how varying one or more components influenced outcomes over a period of time. As described in greater detail below, simulations were conducted to examine how work disability-related management strategies implemented by both companies impacted RTW. Using the simulation model, preliminary model sensitivity tests were also conducted. Component values were set to extreme conditions to determine if changes to RTW occurred as expected [25, 28].

## Results

### Description of Model Structure

**Fig. 1:**
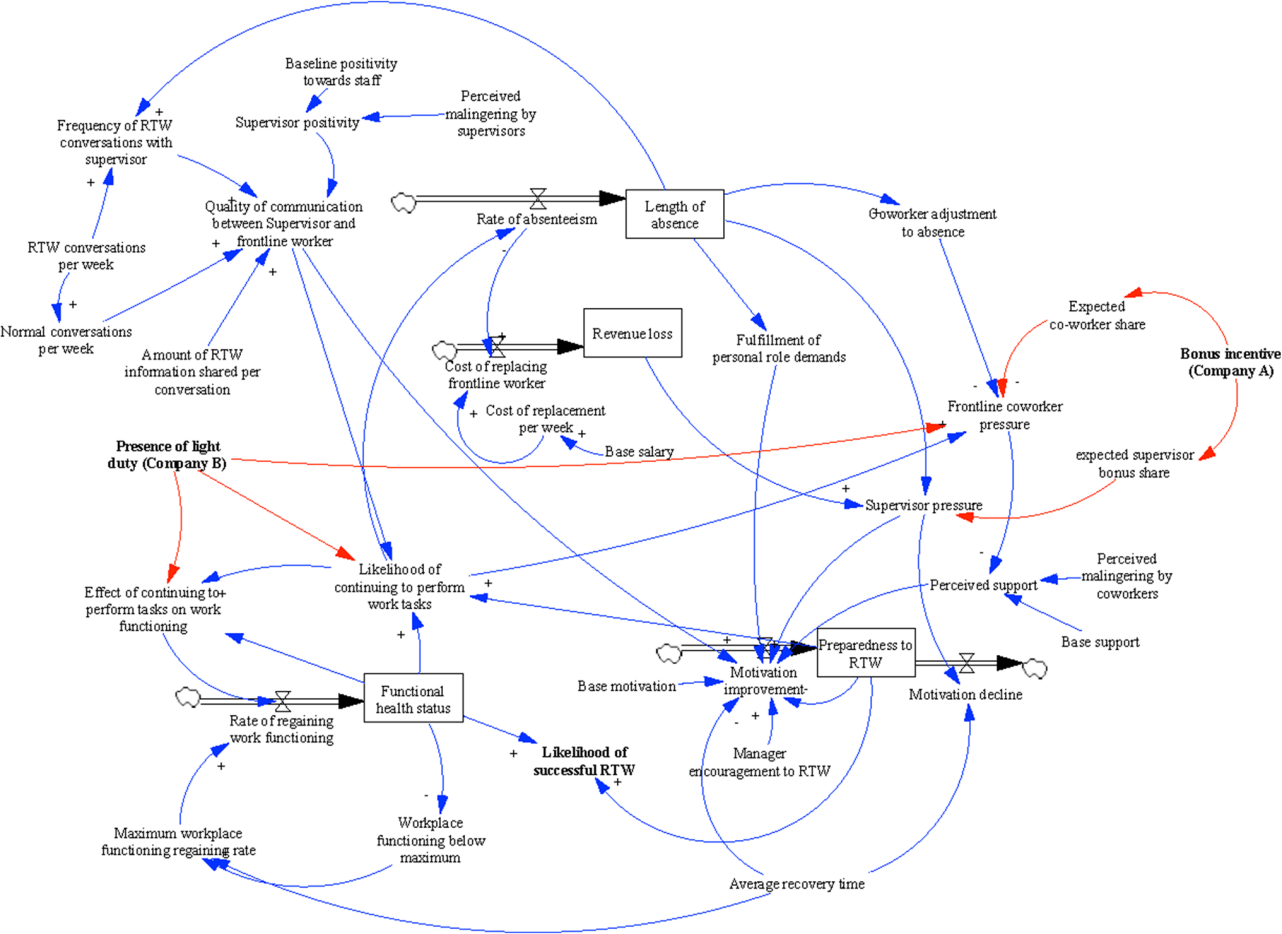
Organizational system dynamics model of the return-to-work process. *Notes*: *Rectangle box* indicates stock variable that accumulates or depletes over time; *Thick arrows* indicates a flow variable which refers to the rate of change in the stock over time; *Plus symbol* a positive relationship which indicates that components change in the same direction; *Minus symbol* a negative relationship indicates that components change in different directions

The SDM presented in Fig. 1 represents the causal loop structure of the organizational work disability systems that emerged from model building sessions. As expected, the SDM was multidimensional, including attitudinal characteristics of the individual (i.e., motivation to RTW, and preparedness to RTW, and fulfillment of role demands outside of work), health factors (i.e., functional health status, and performance of work tasks), social factors (i.e., perceived workplace support, quality of communication between supervisor and worker, and co-worker and supervisor pressure to RTW), and organizational components (i.e., work disability management policies, and revenue loss). The polarity between related variables was also established during model building and is depicted in the figure using ‘+’ (denotes that components change in the same direction), and ‘−’ (denotes that components change in opposing directions). Consistency between the models obtained in the two organizations enabled the depiction of one common model. Several key features of the model will be described in this section.

First, findings from model building sessions showed that the likelihood of RTW within each organization was influenced by two primary stock (level of outcome) and flow variables (rate of change of outcome). Indicated by their positive polarity, greater levels of functional health status (+) and preparedness to RTW (+), increased RTW likelihood. Stakeholders also identified causal pathways between the level of RTW preparedness, and several factors. Indicated by the negative polarity, increases in supervisor pressure to RTW (−) and role demands outside of work (−) resulted in lower preparedness. Quality of supervisor-frontline worker communication (+), and co-worker social support (+) were components that had an opposing impact, and increased RTW preparedness.

The model building exercise also revealed that quality of communication was increased by greater supervisor positivity (+), frequency of RTW conversations (+), and amount of information shared regarding work injury (+). Additionally, the length of absence was linked to several model components including role demands outside of work (+), coworker adjustment to workplace injury (+), and supervisor pressure on an injured worker to RTW (+).

Despite similarities in terms of components and feedback relationships uncovered through model building, each company implemented unique policies (depicted as red arrows in Fig. 1) to manage work disability-related costs and facilitate RTW. Company A had a financial incentive, offering a $60,000 annual bonus to be divided amongst all workers. When a work injury occurred, money was deducted from the bonus pot to pay for immediate medical care (e.g., emergency room visit, and initial treatment). Within the specific organizational context, the policy was intended to prevent workplace injuries, incentivize safety behaviors, and minimize short-term health care costs. Modeling sessions identified a causal pathway between the bonus pot and pressure frontline co-workers (+) and supervisors (+) placed on injured workers, suggesting that the policy may have had an unintended impact on the workplace social climate.

In comparison, Company B offered light duty. Work disabled employees that were medically cleared for adapted tasks, were found temporary roles that fit their activity limitations. Within the organizational context, light duty aimed at facilitating early work reintegration and minimizing workers compensation costs. Findings from model building uncovered a causal pathway between the presence of light duty and the ability to perform work tasks (+). At the same time, light duty was also related to increased pressure frontline co-workers placed on injured workers to RTW at full duty (+).

### Simulation Scenarios

Next, using the SDM designed in the participating organizations, simulation scenarios were conducted to determine how their unique work disability-related policies influenced the RTW process. The model simulated system behavior over a time period of 24 weeks to capture both simple and prolonged work disability cases. Simulations were examined with respect to their impact on percentage of RTW preparedness (0 = no RTW preparedness; 100 % = completely prepared to RTW) and percentage of RTW likelihood (0 = no likelihood to RTW; 100 % = completely likely to RTW) which were used as proxies for the overall performance of the RTW process [10].

**Fig. 2:**
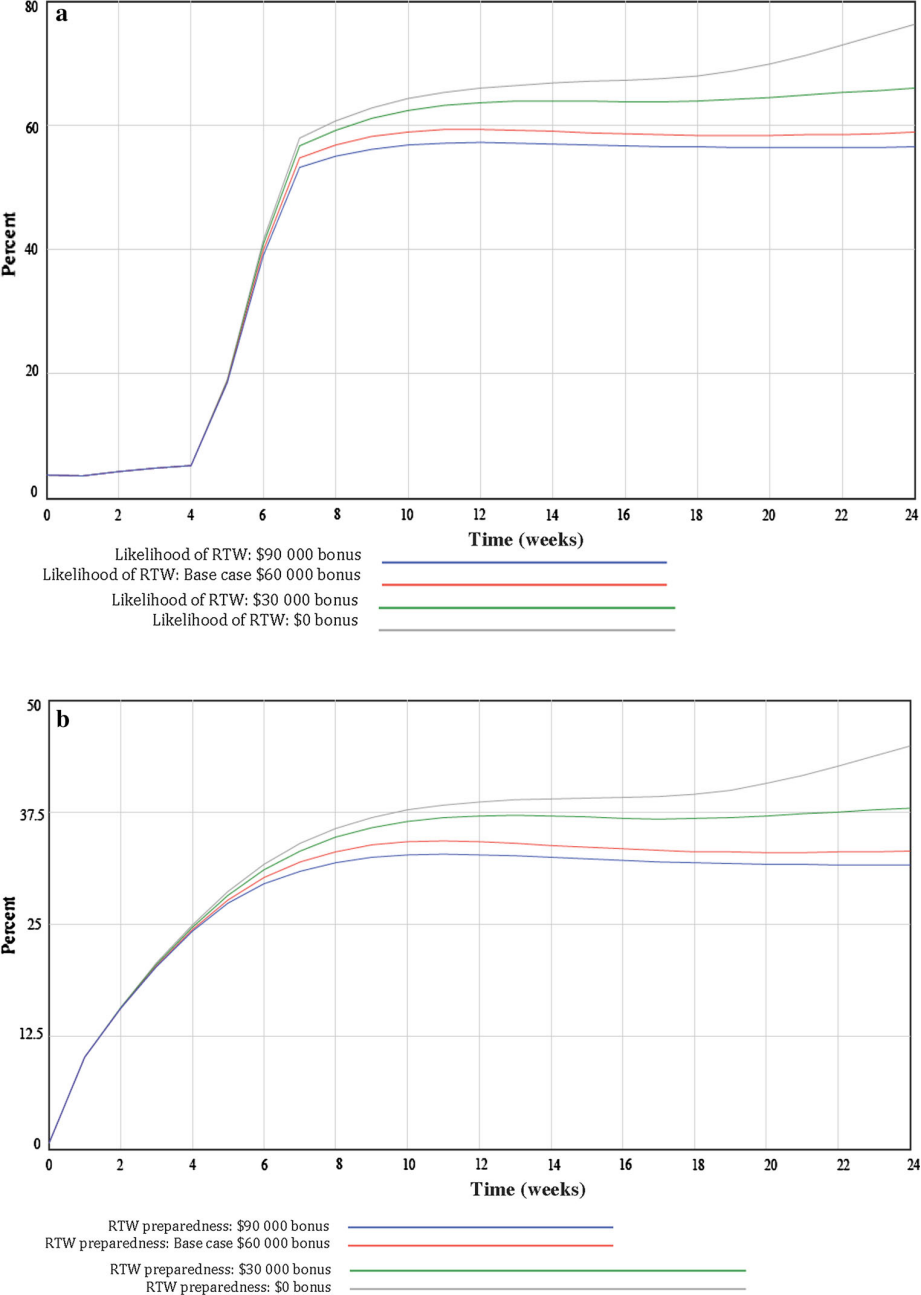

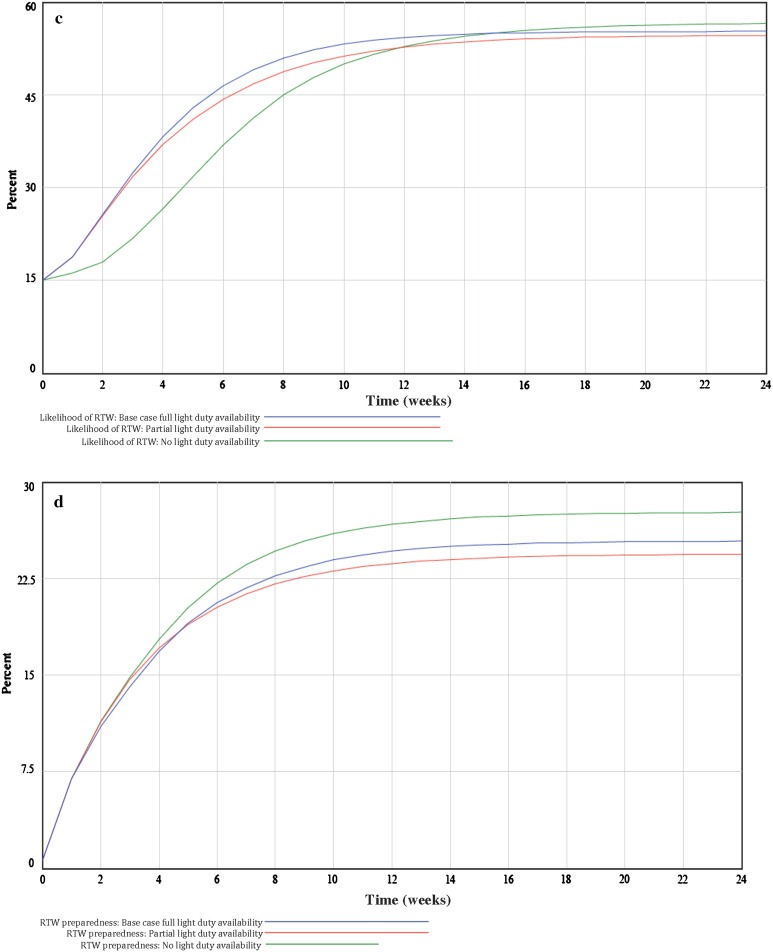
**a** Findings from system dynamics model simulation comparing incentive-based organizational policy over a 24-week time period on the likelihood of return-to-work (RTW). **b** Findings from system dynamics model simulation comparing incentive-based organizational policy over a 24-week time period on return-to-work (RTW) preparedness. **c** Findings from system dynamics model simulation comparing light duty organizational policy over a 24-week time period on return-to-work (RTW) likelihood. **d** Findings from system dynamics model simulation comparing light duty organizational policy over a 24-week time period on return-to-work preparedness. *Notes*: **a**, **b** Comparison of four incentive amounts—$60,000 (base case), $30,000, $90,000 and $0, and preparedness of likelihood of RTW; **c**, **d** Comparison of full (base case), partial, and no light duty and likelihood, and preparedness of RTW

In Company A, where a bonus was provided as an incentive to prevent work injuries, simulations were conducted to compare the current (base case) company-wide bonus ($60,000) to increased ($90,000), decreased ($30,000), and no bonus ($0) scenarios. The simulation presented in Fig. 2a showed that RTW likelihood trajectory was initially low (0–4 weeks), followed by a rapid linear increase, and than a plateau (8 weeks). At first (0–6 weeks), few differences existed between the different bonus levels and the likelihood of RTW (range 39–41 %). At 12 weeks, the differences between the scenarios became apparent. In contrast to what was expected, no bonus (66 %) and reduced bonus (64 %) scenarios exhibited higher RTW likelihood when compared to the current policy (59 %) and increased bonus (57 %) scenarios. The simulation conducted in Fig. 2b found that RTW preparedness increased logarithmically over the time period (Fig. 2b). Few differences existed between the different bonus levels and RTW preparedness between 0 and 6 weeks (range 29–31 %). At 12-weeks, no bonus (39 %) and reduced bonus level (37 %) scenarios exhibited higher RTW preparedness compared to the base case policy (34 %) and increased bonus scenario (33 %). Differences in bonus levels on RTW preparedness plateaued at the 12-week time point, and persisted over the course of the simulation.

In Company B, where light duty was provided to facilitate early work reintegration, the availability of full light duty (base case) was compared to partial and no light duty. The simulations showed that RTW likelihood (Fig. 2c) and RTW preparedness (Fig. 2d) increased logarithmically over the time period. Indicating it’s usefulness to RTW, at 6 weeks, full (46 %) and partial light duty (44 %) availability exhibited a greater likelihood of RTW, compared to no light duty availability (37 %). In more prolonged cases (12 weeks), little difference existed between full (54 %), partial (53 %) and no light duty (53 %) on the likelihood of RTW. When examining RTW preparedness in Company B (Fig. 2d), findings showed that at 6 weeks few differences existed between full (21 %), partial (20 %) and no light duty (22 %) scenarios. At 12 weeks, the no light duty scenario exhibited slightly higher RTW preparedness (27 %) compared to partial (23 %) and full light duty availability (25 %). Differences in the provision of light duty and RTW preparedness also plateaued at 12 weeks and persisted over the remaining 12 weeks of the simulation.

## Discussion

The SDM developed in this study provided a unique view of the RTW process and demonstrated that organizational work disability systems may be viewed as complex, consisting of individual, psychosocial, and organizational components connected by dynamic feedback relationships. Additionally, this study identified potentially critical causal pathways within the system that may explain and quantify how employer-based work disability policies influence RTW trajectories. Characteristic of complex adaptive systems, quantified model simulations found that the implementation of work disability-related policies can result in unanticipated consequences for RTW outcomes. By examining RTW as a system we may inform the ways in which policies and programs are applied within complex organizational systems. More research is required to determine the fidelity of SDM as a research and practice tool in the field of work disability prevention.

SDM offered a lens to capture qualitative and quantitative systematic characteristics of RTW in two organizations. Consistent with previous biopsychosocial models of work disability [4, 5], the SDM included interrelated individual, psychosocial and organizational components. The combination of multiple feedback loops with distinct amplifying or dampening effects on RTW provided a depiction of the non-linearity that underlies complexity, and explains why RTW outcomes can be variable and sometimes difficult to change [19, 29]. Among the causal pathways uncovered in the SDM, specific workplace psychosocial components were identified as being especially important to the RTW process and helped to understand findings from model simulations. Aligning with previous studies [2, 10–12], workplace social support and quality of supervisor-frontline worker communication were components that amplified RTW preparedness. At the same time, co-worker and/or supervisor pressure in response to a workplace injury was an opposing force that dampened RTW preparedness. From a sociotechnical lens, psychosocial components appear to be important leverage points that impact the way a system may respond to policy change [21]. Building a workplace culture that encourages communication and support, and minimizes pressure placed on injured workers can be a systematic strategy that may be taken by an organization to improve RTW outcomes.

Model building also revealed that the two participating organizations differed in terms of the policies they accessed to manage work disability. Policies implemented included an incentive-based preventative policy (Company A) and early light duty provision (Company B). Despite differences in their mechanisms and intended impact, both policies directly effected psychosocial components including supervisor and co-worker pressure on injured workers to RTW. As shown in model simulations, the influence of policies on psychosocial components could potentially result in the emergence of RTW outcomes that were not initially anticipated by employers. Simulations conducted in Company A compared a base case bonus scenario to scenarios with lower and higher bonuses. Findings showed that offering financial incentives decreased RTW preparedness and RTW likelihood. Based on the SDM structure, offering a financial bonus amplified co-worker and supervisor pressure and dampened RTW outcomes, helping to explain the results. This finding is consistent with previous studies examining the use of financial incentives to improve occupational health outcomes [2, 30]. These previous studies showed that incentives could create an environment where a workplace injury or illness is primarily treated as a cost to the organization rather than also focusing on the implications to worker health and well-being. The focus on the financial implications of work disability could have a downstream effect on frontline supervisors and co-workers, and result in a sick worker being pressured to RTW [2]. As a way to improve RTW outcomes, organizations could draw on a systems perspective to consider the broader impact of work disability management policies on all stakeholders within a system, not just injured workers. Findings also suggest that organizations could design policies and programs which consider health and financial goals as equal [12].

Simulations conducted in Company B, examined the impact of light duty availability on RTW. In scenarios where light duty was available, RTW preparedness and likelihood increased. At the same time, the expected difference between the impact of providing light duty versus no light duty on RTW was not as large as anticipated. Using the SDM as a guide, light duty provision might have amplified co-worker pressure and constrained RTW. These findings could suggest that providing light duty to an injured worker may also negatively impact organizational processes (e.g., slowing production process, or minimizing the number of light jobs that are relied upon by non-injured workers for rests during a demanding workday), which may have resulted in the pressure placed on injured workers. Consistent with previous research [13, 17, 18], findings point to organizational RTW outcomes being improved by fostering workplace conditions where both co-workers and supervisors are less adversarial and more supportive, and willing to adapt to changes in the work environment. The minimal effect of light duty might also reflect the various other components that exist in the system and have an opposing influence on the RTW process. By modeling the range of components and the feedback structure of the SDMs, we were able to gain a unique systems view of the organization. This perspective helped to understand why undesirable RTW outcomes may have emerged from the various component interactions, and identified characteristics of the system that could diminish the effectiveness of work disability strategies that were implemented within each company. Insights gathered from the SDM may not be ascertainable through traditional linear models.

As a methodology, SDM has potential implications for managers by providing a tool for system-based decision-making. Through a greater awareness of the multiple feedback relationships that make up the system, decision-makers may be able to better understand more complex work disability problems and consider new creative solutions so that the behavior of the system fits with organizational goals [29]. In-depth knowledge of the system may also enable managers to understand the broader impact of policies on stakeholders and practices within an organization, and predict and manage potential undesired consequences [19, 29]. Additionally, involvement in the model building process may help senior managers and frontline supervisors understand the role they play in the work disability system, and ways in which they can implement changes that improve RTW outcomes. Lastly, by conducting simulations, decision-makers can test dynamic hypotheses and examine how various interventions and changes can impact the system as a whole and visualize anticipated and unanticipated RTW outcomes.

Given its novelty in the field of work disability, the limitations of SDM methodology should be acknowledged. First and foremost encouraging stakeholders to engage in systems thinking and it’s respective methodologies can be challenging, especially for those who view RTW as a step-by-step process [21]. To promote participants to think holistically and consider feedback relationships among components, interview probes were designed to elicit a systems view of the RTW process. Second, engaging different stakeholders with various RTW experiences may result in multiple perceptions of the system. In these situations, the model-builder investigated potential incongruities through additional data collection, and ultimately decided which components were included and how they were related in the model. While there is an element of subjectivity in the approach, findings from our piloting of the methodology showed that by obtaining agreement of the feedback structure during model building sessions, stakeholders confirmed face validity of the SDM, and minimized the need for model builders to impose their views of component inclusion or exclusion [25]. Third, while we took several steps towards establishing preliminary SDM validity, additional research applying models to a greater number of companies is required to examine the applicability of the methodology to a greater number of organizational contexts to, and determine reproducibility. Research is also required to compare simulations to objective RTW outcomes to further establish model sensitivity.

## Conclusion

Complexity is an inherent feature of the RTW process that can underlie variable and often undesirable outcomes. This study was one of the first to apply a sociotechnical systems perspective and SDM methodology to examine complexity in the RTW process, specifically with regard to two industrial companies. Results from model building in both companies showed that individual, social and organizational components and their feedback relationships made up the work disability system and influenced RTW. Psychosocial workplace components could be important leverage points within the system that have the greatest effect on RTW outcomes. The policy-psychosocial component pathway had important implications for the RTW process and may result in RTW outcomes that are not initially anticipated. In sum, a sociotechnical systems perspective provides a unique tool to advance the field of work disability prevention, and inform the ways in which policies and programs are designed and implemented within complex systems.
